# Hybridization but Minimal Introgression: Ecologically‐Based Divergent Selection Maintains a Steep Hybrid Zone in Parapatric Stickleback Fish

**DOI:** 10.1111/mec.70510

**Published:** 2026-08-17

**Authors:** Olivia Baebler, Quiterie Haenel, Krista Oke, Andrew P. Hendry, Daniel Berner

**Affiliations:** ^1^ Department of Environmental Sciences, Zoology & Evolution University of Basel Basel Switzerland; ^2^ Alaska Pacific University Anchorage Alaska USA; ^3^ Department of Biology McGill University Montréal Quebec Canada

## Abstract

Steep hybrid zones provide key insights into the mechanisms of speciation by reflecting incomplete reproductive isolation between diverging populations. However, the specific reproductive barriers preventing the fusion of such populations generally remain unclear, particularly the role of ecologically‐based divergent selection. To address the latter, we investigate a steep zone of transition between lake and inlet stream ecotypes of threespine stickleback fish inhabiting contiguous habitats within a single watershed. Given the spatial proximity of these habitats and the system's postglacial age, historical allopatry is unlikely to have contributed to the evolution of reproductive isolation. Using individual whole‐genome sequencing from clinal sampling sites, we identify ongoing hybridization that is limited to a narrow zone—just a few hundred meters long—around the transition between lake and stream habitat. Individuals in this contact zone exhibit strongly bimodal genome‐wide ancestry, with a rapid shift toward the stream ecotype's genomic background across the lowest stream section, consistent with strong divergent selection and asymmetric gene flow. Individual‐based simulations tailored to this system demonstrate that divergent ecological selection alone can maintain the sharp cline observed and illustrate sustained antagonism between gene flow and selection near the habitat transition. Our findings underscore the power of ecological divergence to generate and maintain reproductive isolation, even in the absence of historical separation, and motivate further empirical work on the ecological underpinnings of steep hybrid zones.

## 1 Introduction

Hybrid zones are geographic regions where genetically diverged groups of organisms meet and produce offspring of mixed ancestry (Barton and Hewitt 1985; Gompert et al. 2017; Stankowski et al. 2021). Most hybrid zones are relatively persistent through time and spatially narrow relative to the dispersal capacity of the organisms involved, hence they likely reflect reproductive isolation between the groups in contact. Because the evolution of reproductive isolation is central to the process of speciation, hybrid zones serve as valuable natural laboratories for studying the origin and maintenance of species (Barton and Hewitt 1985; Jiggins and Mallet 2000; Gompert et al. 2017; Moran et al. 2021).

A central challenge in hybrid zone research lies in disentangling the multiple, potentially interacting components of reproductive isolation. In particular, it is often difficult to assess to what extent hybrid zones are maintained by ecologically‐based divergent selection (i.e., extrinsic reproductive isolation) versus reproductive barriers not directly linked to ecology that evolved during historical periods of physical isolation (Bierne et al. 2013; Harrison and Larson 2016; Gompert et al. 2017; Moran et al. 2021; Stankowski et al. 2021). The latter can include intrinsic postzygotic isolation, arising from the independent accumulation of incompatible genetic variants, or premating barriers such as divergence in mating preferences.

In many taxa, steep and persistent hybrid zones involve lineages that diverged over hundreds of thousands to millions of generations in physical isolation before coming into secondary contact (e.g., Harrison 1986; Szymura and Barton 1991; Bell 1996; Teeter et al. 2008; Smith et al. 2013; Dufresnes and Dubey 2020; Natola et al. 2022; Wang et al. 2022; Kalaentzis et al. 2023; Ebdon et al. 2025; Semenov et al. 2025; but see Stankowski 2013; Westram et al. 2018). While ecological selection may still contribute to reproductive isolation in such cases, the strength and nature of this contribution are often difficult to assess due to confounding with other reproductive barriers (Endler 1977; Barton and Hewitt 1985; Kruuk et al. 1999; Bierne et al. 2013; Moran et al. 2021; Stankowski et al. 2021). A direct opportunity to examine the role of ecological selection, however, arises in hybrid zones involving recently diverged populations where substantial evolution in isolation can be ruled out.

With this opportunity in mind, we here focus on a contact zone between parapatric lake and stream ecotypes of threespine stickleback fish ( *Gasterosteus aculeatus* ). Across the species' Holarctic range, neighbouring lake and stream stickleback frequently show marked phenotypic divergence, reflecting adaptation to limnetic and benthic ecological niches, and this ecological divergence is often accompanied by variable degrees of reproductive isolation and associated genetic differentiation (Reimchen et al. 1985; Lavin and McPhail 1993; Reusch et al. 2001; Hendry and Taylor 2004; Berner et al. 2008, 2009; Deagle et al. 2012; Ravinet et al. 2013; Feulner et al. 2015; Roesti et al. 2015; Moser et al. 2016; Stuart et al. 2017). However, despite their occurrence in close physical proximity and the associated potential for gene flow, little is known about the extent of hybridization between lake and stream stickleback ecotypes at the genomic level.

**FIGURE 1:**
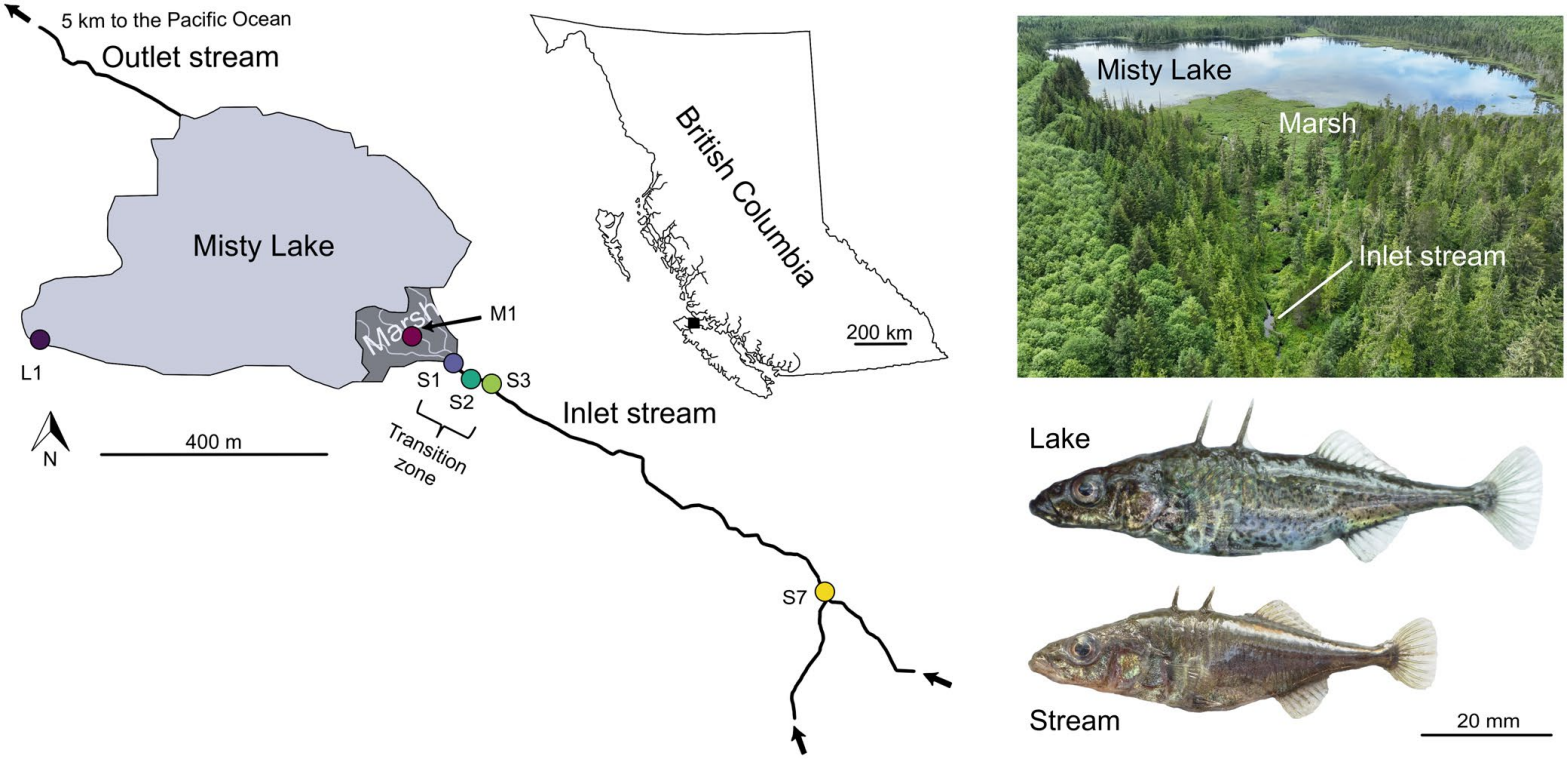
Overview of the Misty Lake and inlet stream system. The dots in the map indicate the lake (L1), marsh (M1) and stream sites (S1‐S3, S7) where the experimental individuals were collected. The insert map shows the location of the Misty watershed on Vancouver Island, British Columbia, Canada. The aerial photograph shows the lake, the marsh, and the lowest reaches of the inlet stream where the genetic transition zone (sites S1‐S3) is located. The stickleback specimens are representative males in breeding dress from the pure lake and stream ecotypes, raised under common‐garden conditions. Maps adapted from Haenel et al. (2021), drone image from Andrew Hendry, stickleback specimens from Daniel Berner.

To address this gap, we conduct a genomic investigation of lake and inlet stream stickleback occurring in the Misty Lake watershed on Vancouver Island, Canada (Figure 1; Lavin and McPhail 1993; Hendry et al. 2002; Moore et al. 2007; Hanson, Moore, et al. 2016). This ecotype pair has been revealed by previous ecological, biogeographic and phylogenetic evidence to be postglacial in origin (< 12,000 generations) and to have diverged in parapatry (i.e., primary intergradation) (Hendry and Taylor 2004; Stuart et al. 2017; Haenel et al. 2021). Experimental crosses between Misty Lake and stream fish reveal no intrinsic postzygotic incompatibilities (Lavin and McPhail 1993; Hendry et al. 2002; Berner et al. 2011; Poore et al. 2023)—a general result for postglacial stickleback populations (Hendry et al. 2009). Moreover, mating trials indicate negligible genetically‐based sexual isolation between the ecotypes (Raeymaekers et al. 2010; Räsänen et al. 2012), and there is no relevant difference in their reproductive timing (Hanson, Barrett, and Hendry 2016). Nevertheless, clinal genomic work based on pooled sequencing uncovered a steep genome‐wide transition in allele frequencies on a small (c. 200 m) spatial scale, coinciding with the stream section immediately adjacent to the transition from lake and marsh to stream habitat (figure 2b in Haenel et al. 2021). This pattern indicates a role for divergent selection in maintaining reproductive isolation. However, the pooled sequencing underlying this initial genomic investigation precluded inferring the extent of hybridization between Misty Lake and inlet stickleback, the degree to which hybridization leads to introgression across the lake‐stream transition, and how introgression shapes patterns of ancestry across the genome—information crucial to understanding the strength and nature of reproductive isolation.

Our present work thus expands the genomic analysis of Misty Lake and stream stickleback by analysing individual‐level whole‐genome sequences from individuals collected across the previously identified transition zone. Our analyses offer clear evidence of hybridization between the lake and stream ecotypes, but at the same time strong genome‐wide restrictions to introgression. We support these empirical findings with individual‐based simulations tailored to the ecological and spatial characteristics of the Misty Lake‐stream system. Together, our results provide compelling evidence for the efficacy of ecological selection in maintaining reproductive isolation in a young hybrid zone.

## 2 Materials and Methods

### 2.1 Study Individuals and DNA Sequencing

The clinal study by Haenel et al. (2021) using pooled sequencing of stickleback collected during the 2016 breeding season from Misty Lake and its inlet stream identified a pronounced shift in genome‐wide allele frequencies occurring over a distance of approximately 200 m in the lowest reaches of the stream (sites S1, S2, and S3 in Figure 1; all site names correspond to those in Haenel et al. 2021). Our present investigation of hybridization and admixture is focused on this “transition zone” between the ecotypes and reuses DNA samples from 20 individuals from each of the three transition zone sites (Table S1; see Haenel et al. 2021 for detailed geographic information, and for specimen sampling and DNA extraction methods). To represent the “pure” lake and stream ecotypes with minimal potential hybrid influence, we also included DNA samples from ten individuals collected from locations distant from the transition zone: one site in the lake (site L1) and another in the upper reaches of the stream (site S7) (Figure 1).

The previous clinal study additionally suggested that the marsh habitat situated between the lake and the inlet stream harbours stickleback genetically slightly differentiated from the true lake population (see also Hanson, Moore, et al. 2016), with this distinctiveness occasionally disappearing due to pulses of intense dispersal from the lake (Haenel et al. 2021). To verify these observations, we additionally included two samples of ten individuals each from the marsh site (M1, Figure 1), one collected in 2016 like the main samples from the transition zone, the other one in 2017. Our main sequencing effort, however, was directed at the transition zone in the lower stream, as this is where the major genetic shift between the ecotypes was observed. In total, the present work comprises 100 individuals characterized in Table S1.

All individuals were initially subjected to whole‐genome sequencing to 101 or 151 bp paired‐end reads without enrichment, using three S4 lanes on the NovaSeq 6000 platform of the Genomics Facility Basel, D‐BSSE, ETH Zürich. For 12 individuals, this sequencing effort yielded a read depth substantially below our target of 20×. DNA from the latter individuals was therefore enriched through ten amplification cycles and sequenced to 250 bp paired‐end reads on an SP flow cell (details provided in Table S1). Final median genome‐wide read depth ranged from 10 to 45× across individuals, with a grand median of 29×.

### 2.2 Marker Ascertainment and Individual Allele Counts

Raw sequence reads were aligned to the fifth‐generation assembly of the stickleback genome (449 Mb; Nath et al. 2021) using NovoAlign (v3.03.00, http://www.novocraft.com/products/novoalign; parameters settings provided in the Supplemental Codes). Alignments were converted to BAM format using the R package *Rsamtools* (v2.10.0; Morgan et al. 2022), and nucleotide counts were generated at all genome‐wide base positions with the *pileup* function (settings provided in the Supplemental Codes). Using the alignments, we determined the sex of each individual by dividing the number of reads mapping to the X chromosome (Chr19) by the number of reads mapping to an autosome of similar length (Chr20). This ratio ranged between 0.99 and 1.04 in the homogametic females and between 0.57 and 0.60 in the heterogametic males.

The ascertainment of single‐nucleotide polymorphism (SNP) markers was initiated by reusing pooled sequencing data from Haenel et al. (2021) from the lake site L1 and the stream site S7, also aligned as described above. These datasets are derived from large samples (*n* = 62 individuals for L1; *n* = 50 for S7) sequenced to high read depth (103× and 107×), thus enabling highly robust SNP detection. Two different SNP panels were ascertained. The first, referred to as “ecotype‐distinctive SNPs”, served to achieve high discriminating precision for quantifying genetic structure and individual hybridity and heterozygosity. We here filtered for positions with read depths between 50 and 200× within the lake and the stream sample to exclude genome regions potentially affected by sequencing or mapping artefacts (e.g., repetitive or highly divergent regions), and exhibiting an absolute allele frequency difference (*AFD*; Berner 2019) of at least 0.85 between the samples. This *AFD* threshold, capturing positions where pure lake and stream fish were highly differentiated (albeit generally not fully fixed for alternative alleles), was chosen to balance physical marker resolution and ecotype specificity. As a robustness check, key analyses were repeated with a more stringent *AFD* threshold of 0.95. Obtaining very similar results throughout (e.g., Figure S1), we report only results based on the 0.85 threshold. For the second SNP panel, used primarily for the analysis of local ancestry, we applied the same read depth but no *AFD* filter, thus including any level of lake‐stream differentiation. These “random SNPs” were still required to exhibit a global minor allele frequency of at least 0.05 across the two pools to minimize sequencing artefacts. At both the ecotype‐distinctive and random SNPs, nucleotide counts for both alleles were performed for all individuals. After excluding SNPs located outside the 20 autosomes (i.e., on the sex chromosomes, unassigned scaffolds, or mitogenome), these allele count data comprised 14,929 and 3.84 million SNPs for the ecotype‐distinctive and random marker panels, respectively.

### 2.3 Analysis of Genetic Structure

To gain first insights into the potential hybrid nature of stickleback in the transition zone, we characterized the genetic structure among our study individuals based on a phylogram and an analysis of global ancestry. For the phylogram, we derived a haploid FASTA file from the allele counts at the ecotype‐distinctive SNPs by concatenating for every individual the most common nucleotide at each marker (Berner 2021). Using the R packages *ape* (v5; Paradis and Schliep 2018) and *phangorn* (v2.5.5; Schliep 2011), we then generated a maximum likelihood genealogy based on the most likely substitution model of sequence evolution (TVMe + G, identified using the Bayesian information criterion) and visualized this genealogy as unrooted phylogram.

Global (i.e., overall genome‐wide) ancestry was examined by using STRUCTURE (v2.3.3; Pritchard et al. 2000). Input data were generated by first converting allele counts from the ecotype‐distinctive marker panel to diploid genotype calls for each individual and SNP. For this, we required a read depth of at least 10× but below 2.2× the genome‐wide median read depth and treated variable positions as heterozygous when a binomial test yielded a *p*‐value below 0.01 (a threshold determined by preliminary exploration to optimize the detection of true heterozygotes). SNPs with more than 25% missing data across individuals were excluded, resulting in 14,849 markers. To explore whether global ancestry estimates were robust to the marker panel (ecotype‐distinctive versus random), we used the same genotyping and filtering conventions to additionally generate three replicate input files from the allele counts at the random SNPs, each independently thinned at random to around 15,000 markers to approximate the marker number of the input data derived from the ecotype‐distinctive SNPs. For the ecotype‐distinctive dataset, STRUCTURE was run under the admixture model with correlated allele frequencies across all samples (including the two marsh samples) using *K* = 1–4 populations with 20 replicate runs per *K*, a burn‐in of 100,000 iterations, and 200,000 MCMC iterations thereafter. The most likely number of populations across our individuals was inferred from the mean likelihood of the data given *K* across runs, and the ∆*K* statistic (Evanno et al. 2005; Earl and VonHoldt 2012). Obtaining unambiguous indication of two populations in this way (see below), the analyses based on the random SNPs were performed analogously with *K* = 2 only.

### 2.4 Hybridity and Heterozygosity

Next, we examined global hybridity (hybrid index) and between‐ecotype heterozygosity (hereafter simply “heterozygosity”) for each individual—metrics characterizing the depth of admixture in hybrid individuals (Fitzpatrick 2012; Gompert et al. 2017). Using the diploid genotype data derived from the allele counts at the ecotype‐distinctive SNPs, an individual's hybridity was expressed as the proportion of “stream alleles” (i.e., the alleles in high frequency in the stream ecotype) among the total alleles across all genome‐wide SNPs. Hybridity near zero thus indicated pure lake ecotype individuals and hybridity near one identified pure stream ecotypes, with admixed individuals exhibiting values between these extremes. Heterozygosity was quantified as the proportion of heterozygous markers among all genome‐wide SNPs for a given individual. For this metric, values were expected to be near the maximum of one for F1 hybrids between the pure ecotypes, but lower for later generation hybrids. For visual analysis, we plotted heterozygosity against hybridity for all sample sites, and additionally for just the marsh and the transition zone sites combined. Since the markers in our ecotype‐distinctive SNP panel were not perfectly ecotype‐diagnostic (i.e., not fully fixed for alternative allele between the L1 and S7 pools), we additionally calculated hybridity and heterozygosity for ten synthetic F1 hybrids, serving as an expectation for that hybrid class. These individuals were generated in two alternative ways: first, we used the Haenel et al. (2021) pooled sequencing data to combine at each ecotype‐distinctive SNP a single allele drawn at random from the L1 nucleotide pool according to the observed frequencies of the two SNP alleles, and a single allele drawn similarly from the S7 pool. In a second approach, we formed ten unique L1‐S7 individual pairs based on our individual sequencing data and combined at each ecotype‐distinctive SNP a single allele drawn from each individual's diploid genotype. Because synthetic F1 hybrids derived from pooled versus individual‐level sequencing were visually indistinguishable in their hybridity and heterozygosity, we just present results from the latter approach.

### 2.5 Local Ancestry Inference

While the above analyses provided global (i.e., genome‐wide) insights into hybridization, we additionally took advantage of our whole‐genome sequencing resolution to explore potential consequences of hybridization locally along chromosomes within each individual. For this, we used Ancestry_HMM (v1.0.2; Corbett‐Detig and Nielsen 2017), a program estimating ancestry probabilities at each genomic position for each individual in a set of samples, plus the age of hybridization based on the assumption of a single punctuated hybridization event. Input data were generated from the allele counts at the random SNPs. We here considered only markers without any missing data across all study individuals and exhibiting a minor allele frequency of 0.25 or greater in the combined L1‐S7 sequence pool, the latter ensuring highly polymorphic markers expected to provide robust ancestry information. These SNPs were thinned to ≥ 2 kb physical spacing to reduce ancestral linkage disequilibrium (LD), resulting in 153,065 genome‐wide SNPs. (Strong within‐population LD typically decays over a few kilobases in this species, e.g., Roesti et al. 2015). Because LD‐thinning may influence the age estimation of hybridization, we additionally considered input data based on ≥ 5 kb marker spacing (70,310 SNPs). Ancestral SNP allele counts required by the software were determined for each marker by treating L1 and S7 as reference populations and estimating allele frequencies from their sequence pools. Average global admixture proportions, also required as input information, were available from our STRUCTURE analysis (as performed with the random SNPs). Genetic map distances between adjacent markers were calculated based on their physical distance and assuming a uniform crossover rate of 3.11 cM/Mb (Roesti et al. 2013). The simplifying assumption of a uniform recombination rate across the genome is not expected to materially influence local ancestry inference, nor the age estimation of hybridization (Corbett‐Detig and Nielsen 2017; R. Corbett‐Detig, personal communication). Within‐individual variation in ancestry was visualized by local ancestry probabilities (i.e., homozygous for lake or stream ecotype ancestry, or heterozygous) along the first three chromosomes for selected individuals from all sites.

Focusing on the transition zone only, we next asked if specific genome regions were particularly enriched for stream ancestry, potentially revealing genome regions under selection for stream alleles. The marsh site was excluded from this analysis because the selective conditions in that habitat may differ from those in the stream. Since the transition zone proved to harbour a mix of direct lake migrants (or weakly admixed lake ecotype‐like individuals) and more strongly admixed, advanced‐generation hybrid individuals (see Results), we excluded the former. The rationale was that only in substantially admixed individuals, the opportunity for the selection of localized genomic regions was given. The transition zone individuals considered as admixed and retained for this analysis were required to exhibit a hybridity greater than 0.2 (*n* = 35, Table S1). Across these individuals, we averaged the estimated probability of homozygous stream ancestry for each marker and visualized the resulting values along the first three chromosomes.

To examine a potential footprint of selection in the transition zone more formally, we identified all markers with an exceptionally high (≥ 0.8) average probability for homozygous stream ancestry, and assessed if this small subset of markers (*n* = 736, 0.48% of all SNPs, located on 15 chromosomes) also displayed elevated differentiation (*AFD*) between the L1 and S7 sample based on the pooled sequence data. Such an association would suggest that genome regions harbouring alleles locally favoured in the transition zone (high stream ancestry) tend to coincide with those under long‐term divergent selection between the pure ecotypes (high *AFD* between ecotypes). As a control, we repeated this procedure with markers exhibiting an exceptionally high (≥ 0.5) average probability for homozygous *lake* ancestry, predicting that this SNP subset (*n* = 636, 0.42% of all markers, on 14 chromosomes) reflects genome regions relatively unimportant to local adaptation and hence does not exhibit elevated L1‐S7 pooled sequencing differentiation. The bootstrap distribution for mean ecotype differentiation was determined for each of these two marker categories based on 10,000 resampling iterations. Benchmarks for these analyses were generated by bootstrap resampling the full set of markers used for ancestry analysis 10,000 times to the same size as the high stream and high lake ancestry subsets, and for each iteration's subsets recalculating average ecotype differentiation from the pooled sequencing data. To verify the robustness of the results obtained, we modified this analysis of signatures of selection by using 10 kb sliding windows instead of individual SNPs as data points, the median instead of the mean as location statistic, and more stringent or more liberal ancestry probability thresholds for marker subset selection. None of these modifications led to qualitatively different results (details not presented).

### 2.6 Influence of Recombination Rate Variation on Introgression

Divergent selection between lake and stream stickleback is polygenic (Roesti et al. 2012, 2015; Rennison et al. 2019; Haenel et al. 2021; Laurentino et al. 2020; Poore et al. 2023), and recombination rates in stickleback are relatively consistently and strongly elevated in the chromosome peripheries compared to the chromosome centers (Roesti et al. 2012, 2013). Combined, these conditions may allow for heterogeneous introgression between habitats across the genome (Berner and Roesti 2017; Haenel et al. 2018; Veller et al. 2023). Specifically, under divergence with gene flow and associated admixture, chromosome segments containing locally advantageous alleles may be especially long in chromosome centers where recombination rates are relatively low. In these regions, divergent selection may be particularly effective due to the strong physical linkage of alleles, potentially leading to an enrichment of locally adaptive ancestry in chromosome centers (Roesti et al. 2012; Berner and Roesti 2017; Veller et al. 2023).

We explored such potential influence of broad‐scale heterogeneity in recombination rate on introgression both for the transition zone and for the pure populations. For the former, we averaged individual probabilities of pure stream ancestry at each SNP across all individuals from the transition zone, again considering only the 35 individuals substantially admixed (Table S1). We then calculated mean stream ancestry across all SNP‐specific averages separately for the periphery of each chromosome and for their centers. Following Haenel et al. (2021), we considered the 5 Mb from either chromosome tip as the periphery, and the remaining region as the center, a delimitation that effectively captures broad‐scale heterogeneity in recombination rate in this species (Roesti et al. 2013). Using the 20 autosomes as replicate data points, we then explored differences in stream ancestry probability between chromosome centers and peripheries. For this, we expressed the 95% compatibility intervals for the chromosome region‐specific median ancestry probabilities by the central 95 percentiles of the bootstrap distributions based on 10,000 bootstrap re‐samples of the chromosomes. Because our L1 and S7 samples included only ten individuals, we used differentiation (*AFD*) between the sequence pools along chromosomes instead of local ancestry to examine heterogeneous introgression between the pure ecotypes. Apart from this modification, we followed the same analytical protocol as for the transition zone.

Introgression probability along chromosomes may not only be influenced by heterogeneity in recombination rate, but also by heterogeneity in gene density. Gene‐rich chromosome regions, likely exhibiting an elevated density of selection targets, may be selected for locally favourable ancestry more effectively and hence show relatively reduced introgression. We considered this potential confounding factor by calculating gene density (number of genes per Mb) for the peripheries and centers of all autosomes, based on the gene annotation of the stickleback genome (Nath et al. 2021). This revealed no relevant difference in gene density between stickleback chromosome centers and peripheries (Figure S2), so that we did not further consider this factor in the above analyses.

### 2.7 Individual‐Based Simulations

To improve our understanding of the key determinants of hybridization and admixture at the lake‐stream transition, we complemented our empirical analyses by individual‐based simulations performed with a modification of the stepping‐stone model developed for the Misty Lake and inlet stream system in Haenel et al. (2021). While the original model served to examine changes in pooled allele frequencies along a linear array of adjacent demes, the present implementation aimed to identify combinations of key population genetic parameter values generating genetic signatures in the transition zone similar to the ones observed empirically. The model comprised a total of nine demes, all of which harboured a total of 200 diploid individuals except for the first “lake” deme. The latter was, following empirical evidence (Fisheries and Ocean Canada 2018), modelled *f* times larger than each of the other (“stream”) demes. The first stream deme, directly adjacent to the lake deme, was our focal transition zone deme. Our simulations thus did not explicitly model a marsh site because the selective conditions in the marsh may potentially differ from both the lake and the stream habitats, and because the spatial structure of the marsh deviates from the linear structure of the rest of the stream (Figure 1).

Based on ample evidence of polygenic divergent selection on stickleback in lake and stream habitats, we modelled 100 unlinked biallelic loci under selection. At each locus, one allele was favoured in the lake deme but deleterious in all stream demes, while the opposite was true for the alternative allele. In the beginning of all simulations, the two alleles were sampled at random with the same probability of 0.5 across all individuals and demes to avoid stochastic allele loss during the early generations. The model scenario thus mimicked primary adaptive divergence from standing genetic variation typical of stickleback fish (Jones et al. 2012; Terekhanova et al. 2014; Lescak et al. 2015; Roesti et al. 2015; Bassham et al. 2018; Haenel et al. 2018, 2022; Galloway et al. 2020). An individual's performance within a given deme was determined by its genotype across all loci, assuming a per‐allele (haploid) selection coefficient *s* (additive reduction in performance from the local optimum of 1; multiplicative selection was considered and produced similar results). Each generation involved a migration phase during which each deme sent a fraction *m* of its individuals drawn at random into each adjacent deme. This was followed by a phase of selection and reproduction during which a deme's offspring cohort was generated by drawing two individuals from the parental cohort at random, but weighted by their performance, to produce a single offspring until initial deme size was re‐established. Individuals were here treated as hermaphrodites and allowed to mate multiple times. All simulations were run over 1000 generations, although the model already reached migration‐selection equilibrium after around 500 generations (Figure S3).

To keep the simulation effort within reasonable limits, we initiated our analysis by a preliminary series of runs in which we haphazardly combined model parameter (*f*, *s* and *m*) values and looked for patterns of hybridity and heterozygosity in the transition zone deme qualitatively resembling the ones observed empirically for the real transition zone. This preliminary screen (details not presented) identified two parameters as particularly relevant: the scaling factor of the size of the lake deme relative to the stream demes, as well as the migration proportion (i.e., the parameters *f* and *m* described above). We thus assumed a fixed selection coefficient *s* of 0.005 found previously to reproduce realistic allele frequency differentiation for the Misty Lake and inlet stream stickleback system (figure 6b in Haenel et al. 2021) and explored the other two parameters in detail by using Approximate Bayesian Computation (ABC). For this, we performed 300 runs with our simulation model, each time using a unique combination of parameter values drawn at random from uniform distributions bounded between 2 and 50 for *f* and 0.01–0.2 for *m*. With the largest parameter values considered, the model thus comprised 11,600 total individuals, and 20% of a deme's individuals migrated into each adjacent deme (i.e., 40% emigration for the non‐terminal demes, 20% for the terminal ones).

At generation 1000, we characterized the transition zone deme after migration but before reproduction (i.e., the parental cohort, corresponding to our empirical samples). We here took a subsample of 20 individuals to match our empirical sample size, calculated median hybridity and heterozygosity for the five individuals with the highest and lowest values for each variable plus for the subsample as a whole, and saved the resulting six statistics from each run along with the associated values for *f* and *m*. Next, we calculated the same hybridity and heterozygosity statistics for the real sample from site S1, serving as target values for ABC estimation. Finally, we ran 100 replicate ABC estimation runs using the R package *abc* (v2.2.2; Csilléry et al. 2012) with the neural network algorithm and a tolerance of 0.1 to identify values of *f* and *m* for which the simulated statistics best approximated the empirical targets (“estimation optima”). In addition, we ran our simulation model with the most plausible *f* and *m* values identified in this way in ten replicates to quantify patterns of hybridity and heterozygosity at generation 1000, both after migration but before reproduction (parental cohort), and after reproduction but before migration (offspring cohort). This allowed us to appreciate changes in genetic composition occurring within a single generation. Finally, to explore the influence of the *f* and *m* parameters in isolation, we ran simulation series in which we varied just one of these parameters across five levels (*f* = 3, 6, 12, 25, 50; *m* = 0.01, 0.02, 0.05, 0.1, 0.2) while keeping the other parameter at ABC optimum. Unless specified otherwise, all analyses, graphing and simulations were implemented with the R language (R Core Team 2024).

## 3 Results

### 3.1 Transition Zone and Marsh Are Influenced by Both Dispersal and Hybridization

**FIGURE 2:**
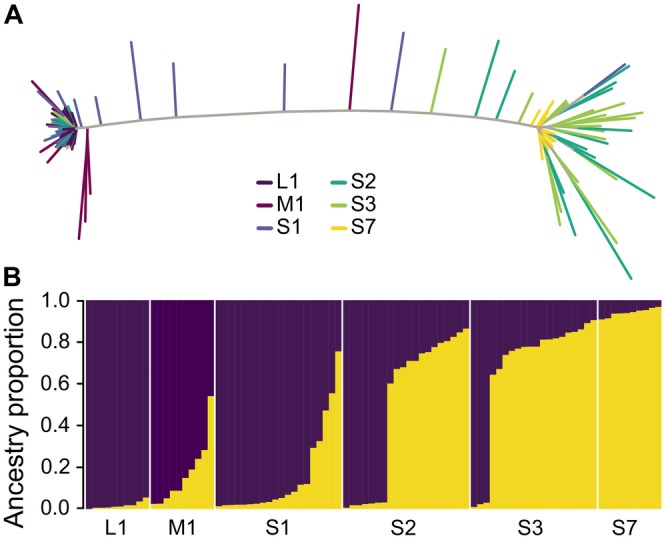
Population structure among stickleback from the Misty Lake and inlet stream system, based on the ecotype‐distinctive marker panel. In (A), individuals are visualized in an unrooted phylogram, while (B) shows their global ancestry proportions when assuming two populations (*K* = 2). Individuals (represented by columns) are sorted by increasing stream ecotype ancestry proportion (yellow) within each sample site. Colour code follows Figure 1.

The phylogram based on the ecotype‐distinctive SNPs revealed that the vast majority of the 60 total individuals collected from the transition zone grouped together either with the pure lake fish (most of the S1 individuals), or with individuals from the upper stream (the majority of the S2 and S3 samples), thus indicating strong bimodality in genomic composition within this stream section (Figure 2A). A few individuals from the transition zone, however, branched between these two deeply separated groups, indicating the presence of hybrids. Such hybrid individuals were also observed at the marsh site, which generally resembled the stream site S1 in genetic composition.

Global population ancestry analysis complemented these tree‐based insights by confirming the presence of two distinct genetic populations (Figure S4) in the Misty Lake‐inlet stream system (consistent with previous microsatellite‐based result; Moore et al. 2007; Hanson, Moore, et al. 2016), and of a small number of admixed individuals with intermediate ancestry (around 0.5; Figure 2B). The latter proved largely restricted to the marsh and the lowest stream site S1; most transition zone individuals either exhibited an ancestry composition similar to the lake fish or approached the ancestry composition seen at the upper stream site. By locating the major shift in ancestry upstream of S1, the global ancestry analysis also highlighted asymmetry in dispersal and admixture in this lake‐stream system. Using the random SNPs instead of the ecotype‐distinctive ones for global ancestry inference produced very similar results supporting the same conclusions (Figure S5).

### 3.2 Marsh and Transition Zone Harbour a Swarm of Deeply Admixed Hybrids

**FIGURE 3:**
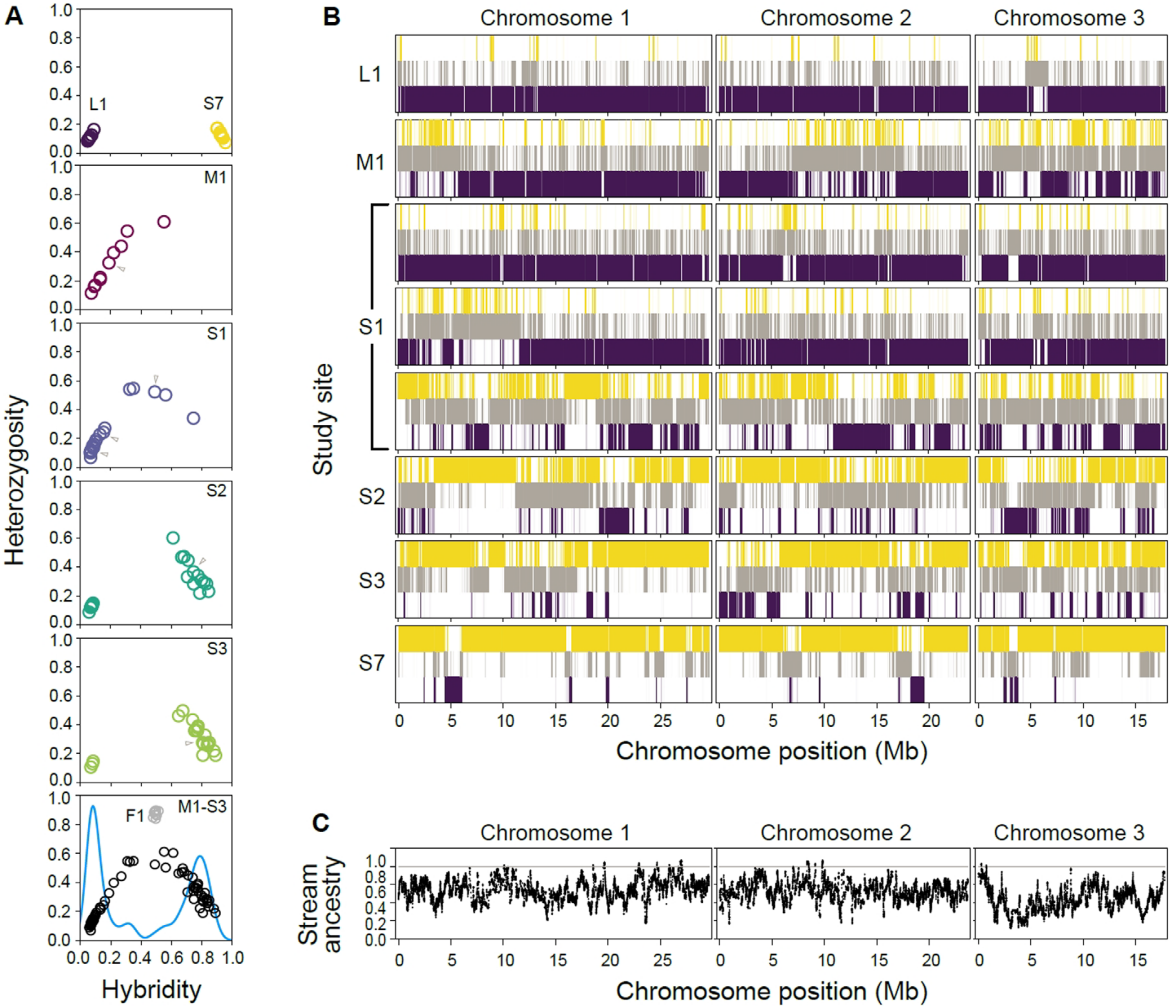
(A) Individual hybridity and between‐ecotype heterozygosity at the sample sites, based on the ecotype‐distinctive SNPs (colour code follows Figure 1). Hybridity represents the proportion of stream alleles across all markers, while heterozygosity expresses the proportion of SNPs heterozygous for lake and stream alleles. The small grey arrows indicate the marsh and transition zone individuals for which local ancestry along chromosomes is presented in (B). The bottom panel depicts all individuals from the marsh and transition zone together, with hybridity kernel density‐smoothed (blue curve) to highlight bimodality. The same panel additionally indicates values expected for F1 hybrids between pure lake and stream ecotypes, based on ten synthetic hybrid individuals (grey circles). (B) Local ancestry along three representative chromosomes, as inferred from the random SNP panel. Probabilities of homozygous lake and stream ecotype ancestry (colour‐coded as in Figure 1) or heterozygous ancestry (grey) are presented for a pure lake and stream individual (top and bottom row), and for selected individuals from the marsh and transition zone (middle rows). For the site S1, a lake disperser, a weakly admixed and a strongly admixed individual are shown. (C) Probability of homozygous stream ancestry averaged over the 35 transition zone individuals exhibiting substantial admixture between the ecotypes (hybridity > 0.2; see A), shown for the same chromosomes as in (B). The grey horizontal line indicates the threshold chosen to delimit markers with an exceptionally high stream ancestry probability examined for a collective signature of selection.

Given clear indications of hybridization in the marsh and the transition zone, we aimed to examine the depth of genomic admixture resulting from hybridization, and the consequences of hybridization to ancestry at the chromosome scale. To initiate the former, we plotted individual heterozygosity against hybridity based on the ecotype‐distinctive SNPs. This polarized the pure lake and stream ecotypes to opposite hybridity, and both groups exhibited low heterozygosity (Figure 3A, top), as expected for markers strongly but not perfectly ecotype‐diagnostic. At the marsh and all transition zone sites, we observed individuals genetically resembling the pure lake fish (delimited by a hybridity < 0.2; Figure 3A, middle rows). Across the transition zone, the fraction of these individuals declined sharply from 75% (S1) to 35% (S2) and 15% (S3), and they were potentially male‐biased; only a third (eight out of 25) were females, although statistical precision was low (bootstrap 95% CI for female probability: 0.15–0.54). The sites M1 and S1 harboured a few individuals with intermediate hybridity near 0.5, whereas most individuals from the sites S2 and S3 tended toward the signature of the stream ecotype. Across the marsh and the transition zone as a whole, hybridity was thus strongly bimodal, and individuals consistent with F1 hybrids (heterozygosity close to one) were notably absent (Figure 3A, bottom).

These observations based on allele frequencies at the ecotype‐distinctive SNPs suggested relatively deep admixture (i.e., the mixing of the pure ecotypes' genomes over multiple generations) for most individuals from the marsh and the transition zone. This was examined further by inferring local ancestry along chromosomes from the random SNPs, revealing relatively homogeneous genome‐wide ancestry in fish from L1 and S7 (Figure 3B, top and bottom rows). A large fraction of individuals from the marsh and transition zone, in particular from S1, showed ancestry patterns resembling those from the pure lake sample (Figure 3B, third row), clearly identifying them as migrants from the lake. The other fish from the transition zone generally exhibited numerous ancestry shifts along their chromosomes (Figure 3B, rows 5–7), consistent with a deep admixture history involving extensive recombination between typical lake and stream ecotype chromosomes. Individuals from the transition zone were indeed estimated to originate from admixture pulses between the pure lake and stream ecotypes having occurred between 151 (S2) and 322 (S1) generations ago. This result was somewhat contingent on our marker thinning; with 5 kb‐thinning, the pulses were estimated between 64 and 140 generations in the past. Irrespective of the details, both sets of analyses unequivocally indicated sustained admixture over numerous generations in the marsh and the transition zone. With continuous migration—violating the assumption of a single hybridization pulse—admixture time tends to be underestimated (Corbett‐Detig and Nielsen 2017), hence our conclusion is conservative. Interestingly, a few individuals from M1 and S1 with slightly higher hybridity than typical lake migrants (around 0.15–0.2) showed distinctive tracts of extended heterozygous ancestry on multiple chromosomes (Figure 3B, rows 2 and 4; further examples from S1 are presented in Figure S6), suggesting recent backcrossing of admixed individuals to lake migrants.

### 3.3 Signature of Selection in the Transition Zone

**FIGURE 4:**
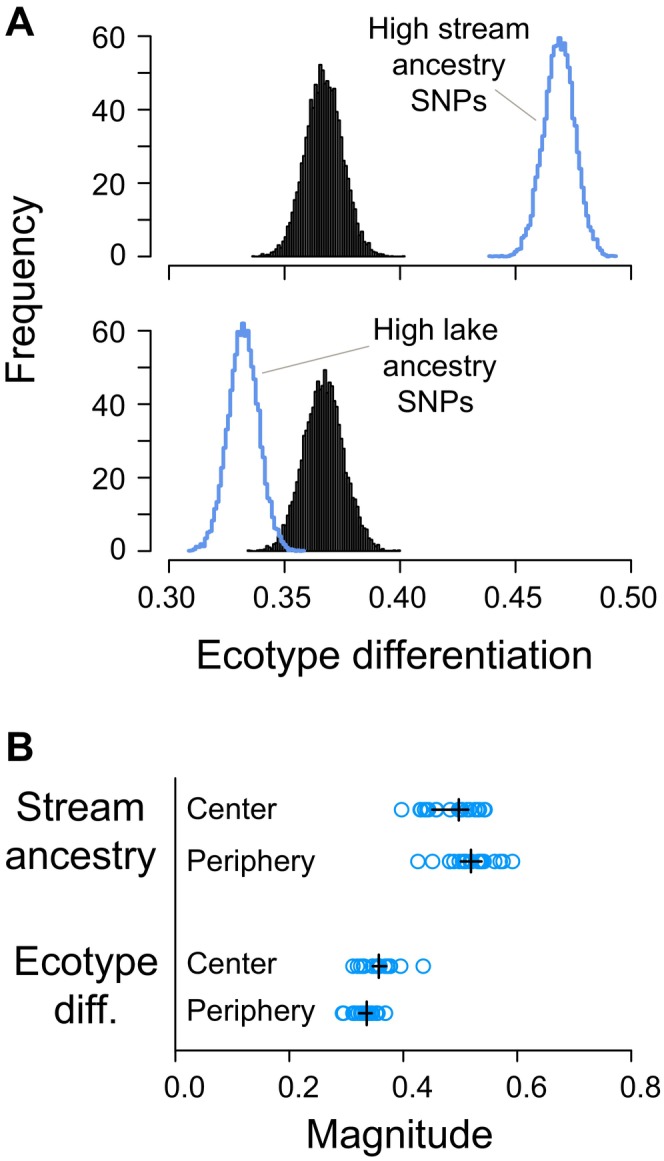
(A) Signature of selection in the transition zone. The blue curves indicate the bootstrap distribution for the mean magnitude of genetic differentiation (expressed by the absolute allele frequency difference *AFD*) between the pure lake and stream ecotype pools for markers showing an exceptionally high probability of homozygous stream ecotype ancestry (top, *n* = 736 SNPs, see Figure 3C) or lake ecotype ancestry (bottom, *n* = 636 SNPs) in the transition zone. Only individuals with substantial admixture (hybridity > 0.2) were considered (see Figure 3A). As baseline for comparison, both graphs include the bootstrap distribution of the analogous statistic for SNPs with random ancestry. (B) Check of an influence of heterogeneity in recombination rate (low in the chromosome centers, high in their peripheries) on the probability of homozygous stream ecotype ancestry in the transition zone (top), and on the magnitude of genetic differentiation (*AFD*) between the pure lake and stream ecotype pools (bottom). The blue circles are chromosome‐specific means, the black vertical lines are their medians, and the black horizontal lines are 95% bootstrap compatibility intervals for the latter based on 10,000 chromosome resamples.

To obtain evidence of selection in the transition zone, we inspected average local ancestry probabilities along chromosomes across the 35 individuals proving well‐admixed (hybridity > 0.2). This revealed high heterogeneity in ancestry probabilities (Figure 3C), and a clear footprint of selection: markers exhibiting exceptionally high average stream ancestry probability in the transition zone also showed substantially (28%) elevated genetic differentiation between the pure lake and stream ecotype samples shaped by long‐term selection (mean *AFD* = 0.468), compared to marker samples of similar size drawn at random (resampling mean *AFD* = 0.367) (Figure 4A). Conversely, markers exhibiting a high average *lake* ancestry probability in the transition zone displayed 10% lower average L1‐S7 differentiation (*AFD* = 0.332) than random marker samples, consistent with these loci being relatively unimportant to adaptive divergence. Neither ancestry probabilities in the transition zone nor the magnitude of differentiation between the pure ecotypes proved substantially influenced by large‐scale heterogeneity in recombination rate, as quantified by the central versus peripheral position of markers along chromosomes (Figure 4B; genomic differentiation between the pure ecotypes is characterized in detail in figure S2 of Haenel et al. 2021).

### 3.4 Simulations Support Asymmetric Ecotype Population Sizes and Massive Lake Dispersal Into the Lower Stream

**FIGURE 5:**
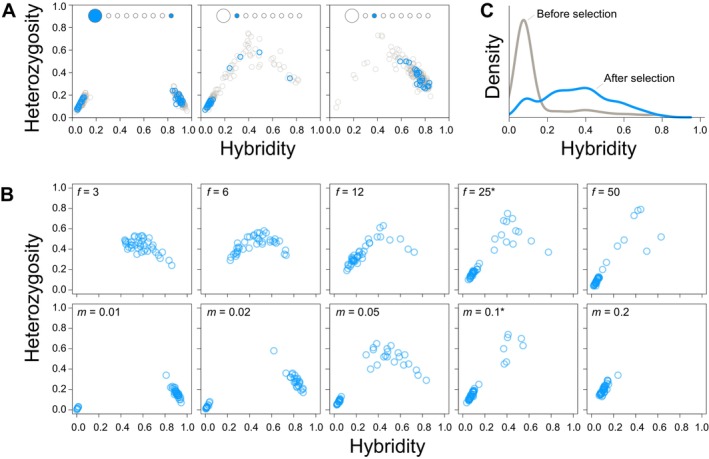
Individual hybridity and heterozygosity in the stepping‐stone simulation model at migration‐selection equilibrium. In (A), results based on ABC parameter optima for the deme size scaling factor (*f* = 28) and the migration rate (*m* = 0.09) are shown for individuals from the lake and the terminal stream deme (left), and for the first and second stream deme (middle and right). The position of the demes in the model is illustrated schematically at the top of each panel. The grey data points represent a pool (*n* = 200) of 20 randomly chosen individuals from each of the ten replicate simulation runs, while the blue data points show a random sample of 20 individuals from a single replicate. (B) Influence on the genetic composition in the first stream deme of varying the *f* or *m* parameter in isolation (i.e., keeping the other parameter at ABC optimum). The data points are 40 random individuals from a single simulation run. The parameter values flagged by an asterisk are those closest to the ABC optimum. (C) Change in hybridity within a single generation in the first stream deme. The curves reflect kernel density smoothed relative density, not absolute individual number (which is much higher before selection due to immigration). Peak hybridity is low before selection because most individuals are fresh immigrants from the lake but rises strongly due to selection.

Our empirical analyses above indicated a key role of dispersal and divergent selection in the Misty Lake‐inlet stream system. To explore whether observed patterns of admixture can arise just from a simple antagonism between dispersal and selection, we combined individual‐based stepping‐stone simulations with Approximate Bayesian Computation (ABC). This revealed that patterns of hybridity and heterozygosity best approximating our empirical results could be reproduced with a model involving a lake deme much (28×) larger than the stream demes, combined with a high migration rate (around 0.09) among adjoining demes (Figure S7). Simulations with these optimal parameter estimates indeed produced (1) strong local adaptation in the terminal demes of the model (Figure 5A, left); (2) a first stream deme dominated by lake migrants but exhibiting a minor fraction of highly admixed individuals (Figure 5A, middle); and (3) a strong shift toward predominant stream hybridity in the second stream deme (Figure 5A, right) (compare to rows 1, 3 and 4 in Figure 3A). Note that the model allowed for dispersal between neighbouring demes only, hence stream demes beyond the first one lacked the direct lake migrants observed empirically at the sites S2 and S3. Modelling weaker asymmetry in deme size prevented strong local adaptation in the lake deme, and hence the possibility of dispersal of lake individuals with low hybridity into the first stream deme (Figure 5B, upper row). Modelling a lower dispersal rate in turn reduced the probability of hybridization and the associated occurrence of admixed individuals in the first stream deme (Figure 5B, lower row). With an extremely high dispersal rate, however, the first stream deme became overwhelmed by dispersers from the lake, thus impeding local adaptation and hence admixture.

Our model with ABC parameter optima further highlighted massive oscillation in the genetic composition of individuals in the stream deme adjacent to the lake during each generation. Due to the large size of the lake deme relative to the stream demes, dispersal caused around 97% of the individuals in the model's first stream deme to be direct dispersers from the lake with minimal hybridity (Figure 5C). However, the hybridity of the offspring cohort, that is, after the selection phase, proved greatly shifted upward, thus implying an extremely low local reproductive contribution of the numerically dominant lake migrants across generations.

### 3.5 Temporal Change in Admixture in the Marsh Habitat

**FIGURE 6:**
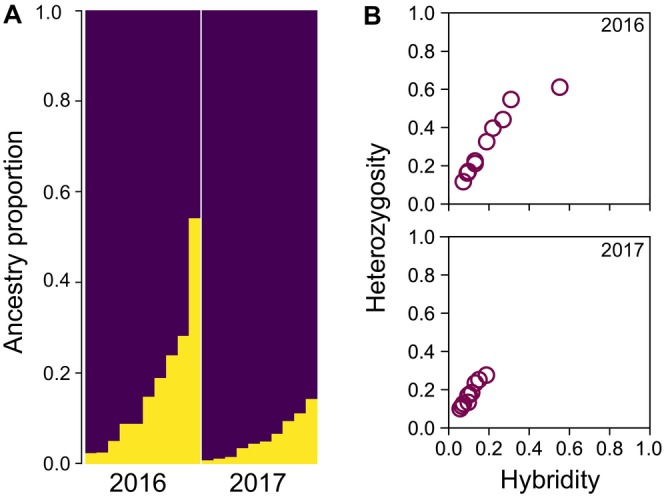
Change in genetic composition at the marsh site (M1) sampled in two consecutive years, as revealed by (A) global ancestry proportions inferred from the ecotype‐distinctive SNPs and considering all sample sites in the same analysis, and (B) hybridity and heterozygosity.

Previous work indicated close genomic similarity between the marsh stickleback and the lake ecotype, but also that allele frequencies in the marsh can change rapidly (Haenel et al. 2021). We here took the opportunity to examine whether this was mirrored by temporal changes in admixture based on individual‐level sequence data from two subsequent years. We found that while in 2016—our main sampling year—individuals from the marsh resembled individuals from the neighbouring stream site S1 in their global ancestry proportion (Figure 2B), the 2017 marsh sample showed a marked shift toward the lake population (Figure 6A). A strong shift toward the genomic composition of the pure lake fish was also evident from individual hybridity and heterozygosity, with all marsh individuals from 2017 exhibiting hybridity below 0.19 (Figure 6B). These patterns are consistent with a pulse of immigration from the lake into the marsh between the sampling years, a view that was refined by local ancestry analysis: the chromosomes of the 2017 marsh individuals were strongly dominated by tracts of homozygous lake ancestry, with few windows of extended heterozygous or homozygous stream ancestry (Figure S8), consistent with the backcrossing of 2016 marsh residents with immigrants from the lake.

## 4 Discussion

Previous work on Misty Lake and inlet stream stickleback based on pooled sequencing identified a sharp transition in marker allele frequencies in the lowest reaches of the inlet stream (Haenel et al. 2021), raising a question crucial to understanding reproductive isolation in this system: to what extent does this genomic transition reflect hybridization and associated admixture, as opposed to just dispersal causing genetically differentiated, non‐admixed ecotypes to co‐occur? Our present investigation of the transition zone based on individual‐level sequence data resolves this question by revealing the presence of both processes—hybridization and dispersal—thereby shedding light on mechanisms of reproductive isolation in parapatry.

As indicated by phylogenetic and global ancestry inference, the transition zone harbours a small fraction of individuals genomically intermediate between the lake and the stream ecotype, and a larger fraction of individuals displaying a proportion of stream ancestry approaching—yet still substantially below—the one seen in the pure stream ecotype sample. All these individuals are clearly admixed, hence derive from hybridization. At the same time, we find a substantial fraction of individuals genomically very similar to, or indistinguishable from, the lake ecotype, which we interpret as direct dispersers from the lake. This dispersal is highly restricted spatially, as we observe a strong decline in the fraction of lake dispersers from the site S1 to S3, that is, over a stream section of just around 90 m (Figure 1). That lake dispersal may indeed not reach much beyond the transition zone is consistent with the absence of pooled allele frequency changes across the whole stream section from a site located around 80 m upstream of our site S3 up to the site S7 (Haenel et al. 2021).

More nuanced insights into the depth of admixture around the lake‐stream habitat transition were obtained by quantifying hybridity and heterozygosity, and by estimating local ancestry along the chromosomes. These analyses clearly confirm that the marsh and transition zone sites combined are composed of two main classes of individuals: immigrants from the lake, and a swarm of deeply (over many generations) admixed hybrids with predominant stream ancestry. Hybridization involving pure lake fish appears to occur in immediate proximity to the lake‐stream habitat transition only (sites M1 and S1); the sites S2 and S3, located just a few dozen meters further upstream, harbour no individuals of intermediate hybridity. Across the latter section of the transition zone, admixed individuals must thus interbreed, but not backcross with the pure lake immigrants, or at least such backcrossing produces no surviving offspring. Nevertheless, the full convergence of the fish in this zone toward the pure stream ecotype seems constrained by the continuous immigration of individuals originating from hybridization with lake fish slightly further downstream. Because individuals qualifying as pure stream ecotypes are absent across the entire transition zone, F1 hybrids between the pure ecotypes are never produced in this lake‐stream system.

The genomic bimodality seen across the marsh and transition zone is a clear indication of strong reproductive isolation driven by divergent selection against genomically intermediate individuals (Barton and Hewitt 1985; Jiggins and Mallet 2000; Stankowski et al. 2021). Sexual reproductive barriers are unlikely to contribute to this bimodality, as experimental evidence reveals mating isolation between the Misty Lake and stream ecotypes and their hybrids to be weak at best (Raeymaekers et al. 2010; Räsänen et al. 2012; see also Irwin 2020). Strong divergent selection and the associated reproductive isolation between the lake and stream ecotypes may actually explain the absence of sexual isolation between the ecotypes. Given strong selection against the lake genome in the stream, avoiding hybridization through sexual premating isolation would appear selectively favourable, but the evolution of such a sexual barrier (i.e., reinforcement) requires extensive opportunity for mating and hybridization between the pure ecotypes (Coyne and Orr 2004; Servedio and Noor 2003). As our analyses indicate, these conditions are not met; hybridization occurs too rarely and locally in the Misty system as a whole.

Evidence of selection in the transition zone emerges not only from the spatial distribution of ancestries, but is also indicated more directly by the comparison of marker classes differing in average ancestry among the admixed individuals in their magnitude of genetic differentiation between the pure ecotypes. We found that markers relatively enriched for stream ancestry exhibited roughly 40% stronger ecotype differentiation than markers enriched for lake ancestry, indicating ongoing selection of genetic material in the transition zone. Interestingly, neither ancestry probabilities in the transition zone nor the magnitude of differentiation between the lake and stream ecotypes proved materially influenced by heterogeneity in recombination rate along chromosomes, which is known to be strong in stickleback (Roesti et al. 2013). This finding aligns well with theoretical expectations: heterogeneous recombination rate modifies the efficacy of polygenic divergent selection across the genome only when genetic mixing between divergent populations is sufficiently extensive (Berner and Roesti 2017). As implied by the strong bimodality in hybridity across the Misty transition zone, such mixing is largely prevented by divergent selection even in immediate proximity to the lake‐stream transition. In other words, the residence time in the stream of large, locally unfavourable chromosome segments from the lake may be too short for variation in recombination rate along chromosomes to influence introgression probability substantially.

Our empirical results identify dispersal and selection as crucial processes shaping divergence and reproductive isolation in the Misty Lake and inlet system. This is also well supported by our simulations indicating extensive dispersal from a large lake population into the lowest stream section. Despite strong and numerically asymmetric dispersal, the simulations showed that ecologically‐based divergent selection causes nearly complete reproductive isolation between the habitats: successful backcrossing of pure lake deme individuals with admixed individuals occurred only in immediate proximity to the habitat transition, and upstream of that zone, the individuals' genomic composition rapidly approached that of the pure stream deme. This implies an extremely low reproductive contribution of lake migrants in the stream across generations, even in the lowest reaches of the stream where these migrants are numerically dominant. In nature, such selection may plausibly occur via massive genotype‐dependent juvenile mortality, which has been suggested for Misty Lake‐stream stickleback (Hendry et al. 2002) and confirmed for a European lake‐stream stickleback system (Moser et al. 2016) by release‐recapture experiments. Direct insights into how selection occurs across life stages could be obtained by tracking the genetic composition of stickleback cohorts over time around the habitat transition.

Strong reproductive isolation evolved in our simulations without requiring a phase of physical isolation, representing primary divergence from standing genetic variation. The simulations further indicate that antagonism between dispersal and selection, and the concomitant oscillations in genomic composition within generations, can persist as an equilibrium, in line with the deep admixture over many generations inferred empirically for most transition zone individuals. In the long run, this equilibrium may be shifted toward stronger reproductive isolation through the accumulation of additional postzygotic barriers. However, given the slow pace of such mutational divergence (e.g., Bolnick and Near 2005; Hedges et al. 2015) and the absence of irreversibly isolated freshwater species within the genus *Gasterosteus*, Misty Lake and stream stickleback are likely to become extinct or collapse before complete reproductive isolation has arisen (Taylor et al. 2006; Hendry et al. 2009; Behm et al. 2010; Rosenblum et al. 2012; Anderson et al. 2023).

While the main focus of our study was on the genomic transition zone in the stream identified previously, our data from the adjoining marsh habitat suggest that this site too is characterized by antagonism between dispersal and selection. The admixed marsh stickleback must result from interbreeding between the lake ecotype and admixed individuals from the lower stream; hence the lower stream section not only receives immigrant lake ecotypes but also represents a source of dispersal downstream into the marsh. The selective conditions in the marsh are not well understood but may resemble those in the stream. The reason is that the marsh must offer extensive opportunities for benthic foraging, and that selection against lake phenotypes and genotypes has been observed in a release‐recapture experiment using adult marsh fish (Hanson, Moore, et al. 2016). Furthermore, the genomic composition we observed for the marsh samples resembles those obtained for the first stream deme in our simulations performed with the highest dispersal rates (compare Figure 6B to Figure 5B bottom row with *m* = 0.1–0.2). This indicates that the genome of the stream ecotype is selectively favoured in the marsh, but that the recruitment of chromosome segments from the stream ecotype segregating in the lowest stream section is strongly counteracted by immigration from the lake, and by the successful breeding of these immigrants in the marsh. The marsh thus seems generally overwhelmed by gene flow from the lake, although our comparison of marsh samples from two subsequent years shows that this can fluctuate in magnitude, depending on ecological conditions. Indeed, the observed temporal genomic shift toward the lake ecotype can be attributed to strong precipitation right after the 2016 sampling period (but still well within that breeding season) that rose the lake water level, thus flooding the marsh (Haenel et al. 2021). This in turn must have facilitated immigration and reproduction of the lake ecotype in the marsh.

## 5 Conclusion

Lake‐stream stickleback pairs represent iconic examples of parapatric divergence and reproductive isolation, yet patterns of hybridization and admixture across their transition zones have not been scrutinized genomically. Here we demonstrate that despite massive dispersal, admixture between the lake and inlet stream ecotypes in the Misty system is largely limited to a narrow zone around the habitat transition. Strong reproductive isolation has here emerged rapidly and without physical barriers as a side‐product of adaptive divergence, likely promoted by both ecogeographic and genetic factors: on the one hand, the lake‐stream transition is geographically sharp, leading to a steep ecological gradient precluding a niche for phenotypically intermediate hybrid forms (Endler 1977; Nosil et al. 2009). The stream habitat's linear nature further facilitates adaptive divergence (Gavrilets 2004; Gavrilets and Losos 2009). On the other hand, stickleback fish are known for their abundant standing genetic variation, allowing for highly polygenic adaptive divergence and thereby causing reproductive isolation to extend rapidly across the whole genome (Barton 1983; Barton and Bengtsson 1986; Kruuk et al. 1999; Flaxman et al. 2014). Experimental work on hybrid zones in other organisms is needed to address how generally and to what extent divergent ecology is implicated in the buildup and maintenance of steep genomic transitions (Harrison and Larson 2016; Gompert et al. 2017; Moran et al. 2021). It is likely that the rapid emergence of strong reproductive isolation across habitat transitions seen in stickleback fish is unusual, and that steep hybrid zones more typically reflect primarily intrinsic incompatibilities and/or sexual barriers evolved over extended periods of geographic isolation.

## Author Contributions

O.B.: study design; DNA sample curation; analyses and graphing; interpretation of results; writing of a first manuscript draft. Q.H.: all DNA extractions. K.O.: field sampling. A.P.H.: field sampling; funding. D.B.: study initiation, design and supervision; funding; analyses, simulations and graphing; interpretation of results; writing of the final manuscript, with feedback from co‐authors.

## Funding

The study was made possible by financial support from the Swiss National Science Foundation (grant 310030_200374 to DB), and field sampling additionally by Fisheries and Oceans Canada, the British Columbia Ministry of the Environment, and the McGill University Biology Department.

## Ethics Statement

The field samples underlying this study were collected with permission from Fisheries and Ocean Canada, Species at Risk (Licence XRSF 14 2015, File SARA 368), the British Columbia Ministry of Environment (Ecological Reserve Permit 102693), and the British Columbia Ministry of Forests, Lands and Natural Resource Operations (Fish Collection Permit NA16‐225865, File 34770‐20).

## Conflicts of Interest

The authors declare no conflicts of interest.

## Supporting information


**Figure S1:** Consequences on individual hybridity and heterozygosity of using two different *AFD* thresholds for the identification of ecotype‐distinctive SNPs. The left panel shows all 100 stickleback with the *AFD* threshold chosen as standard across the study (14,929 SNPs), whereas the right panel shows the analogous result for a more stringent threshold (501 SNPs). The colour code for the sample sites does not follow the one used across the main paper. Note that both thresholds lead to very similar patterns, apart from the expected nuance that with the 0.95 threshold and hence the markers being more ecotype‐informative, the hybridity range is slightly wider.
**Figure S2:** Gene density in the center versus peripheries of the threespine stickleback chromosomes, expressed as the number of genes per megabase. The chromosome peripheries are defined as the terminal 5 Mb on either side of each chromosome. The blue circles represent values for individual chromosomes, the vertical black lines indicate the median across these values, and the horizontal black lines show the 95% compatibility interval for this median based on 10,000 bootstrap resamples. The compatibility intervals are very similar, indicating no substantial difference in gene density between the two chromosome regions.
**Figure S3:** Establishment of migration‐selection equilibrium during the simulations of lake‐stream divergence with the stepping‐stone model using ABC estimation optima for the *f* and *m* parameters. Shown are the 0.125, 0.5, and 0.875 quantiles (distinguished by increasing colour intensities) for hybridity (purple) and heterozygosity (grey) across 40 individuals sampled from the first stream deme in the model in each generation. Results are shown for three replicate simulation runs. Alleles are initially sampled at random with a probability of 0.5, hence the genetic composition is initially uniform across the entire model. After around 500 generations, the system reaches migration‐selection equilibrium, as revealed by the divergence within this deme between lake‐adapted immigrants and strongly admixed individuals, the latter captured by the top quantiles.
**Figure S4:** Analysis of population structure with the software STRUCTURE, based on the ecotype‐distinctive SNPs and including all 100 individuals from the Misty lake‐stream system. The left graphic shows the likelihood *L(K)* of the genomic data when assuming different numbers of populations (*K*), averaged across 20 replicate runs for each *K*. This demonstrates that assuming a single population across the Misty system is implausible, and that beyond *K* = 2, there is minimal gain in likelihood. The rate of change in likelihood *L'(K)* (right graph) thus clearly reveals two true populations (*K* = 2), as does the ∆*K* statistic derived from this metric (not shown).
**Figure S5:** Global ancestry proportions as estimated by STRUCTURE. The graph follows the conventions of Figure 2B, except that the underlying markers are random SNPs, not ecotype‐distinctive ones.
**Figure S6:** Two individuals from the first stream site (S1) showing distinctive tracts of alternative ancestry along some chromosomes. Specifically, while most chromosomes in these individuals exhibited predominantly homozygous lake ancestry (like in Figure 3B, top row), a few chromosomes displayed extended tracts of heterozygous ancestry (approximately indicated by horizontal blue bars), suggesting relatively recent backcrossing of dispersers from the lake with admixed local individuals.
**Figure S7:** Distribution of Approximate Bayesian Computation (ABC) parameter estimates for the factor *f* determining the size of the lake deme relative to the size of the stream demes (*n* = 200), and for the proportion *m* of individuals dispersing in each generation from a given deme into each neighbouring deme (except for the terminal demes, total emigration is thus 2*m*). The distributions are based on 100 replicate ABC estimation runs, all performed with hybridity and heterozygosity summary statistics from 300 simulations with values of *f* and *m* chosen at random. Shown are median estimates from each estimation run. The grand medians (27.8 and 0.091 for *f* and *m*) were taken as optima for further simulation.
**Figure S8:** Local ancestry along three chromosomes for three exemplary marsh individuals collected in 2017. The selected individuals include the one with the lowest (0.057, top) and the highest (0.187, bottom) hybridity observed in this sample, plus an intermediate one (0.101). All graphing conventions correspond to those of Figure 3B.

**Supplemental Codes:** Supporting Information.

**Table S1:** mec70510‐sup‐0003‐TableS1.txt.

## Data Availability

The raw sequence reads are available from the NCBI sequence read archive under the BioProject accession number PRJNA1218656 (https://www.ncbi.nlm.nih.gov/sra/PRJNA1218656). Individual accession numbers are given in Table S1 in the Supporting Information. A complete compilation of all analytical code is available as Supplemental Codes in the Supporting Information. The full allele count matrix from which all files for analysis were derived is provided on the Zenodo repository (doi: 10.5281/zenodo.20430298).
