# Supplementary material for: Hybridization but Minimal Introgression: Ecologically‐Based Divergent Selection Maintains a Steep Hybrid Zone in Parapatric Stickleback Fish
Source: Mol Ecol. 2026 Aug 17;35(16):e70510. doi: 10.1111/mec.70510 (PMC13482134; doi:10.1111/mec.70510)
Supplement: Supplementary file 1 [file MEC-35-e70510-s003.pdf]

MOLECULAR ECOLOGY

Supplemental Information for:

Hybridization but minimal introgression: ecologically-based divergent selection maintains a steep hybrid zone in parapatric stickleback fish

Olivia Baebler, Quiterie Haenel, Krista Oke, Andrew P. Hendry, Daniel Berner

Figure S1

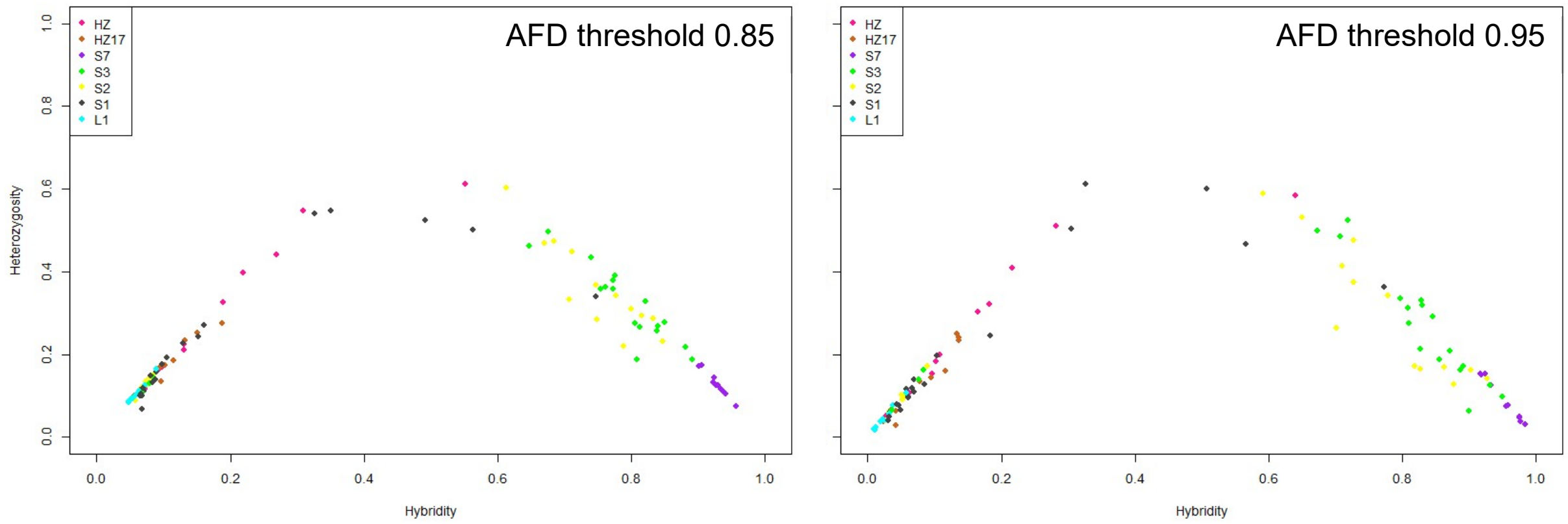

Figure S1. Consequences on individual hybridity and heterozygosity of using two different *AFD* thresholds for the identification of ecotype-distinctive SNPs. The left panel shows all 100 stickleback with the *AFD* threshold chosen as standard across the study (14,929 SNPs), whereas the right panel shows the analogous result for a more stringent threshold (501 SNPs). The color code for the sample sites does not follow the one used across the main paper. Note that both thresholds lead to very similar patterns, apart from the expected nuance that with the 0.95 threshold and hence the markers being more ecotype-informative, the hybridity range is slightly wider.

Figure S2

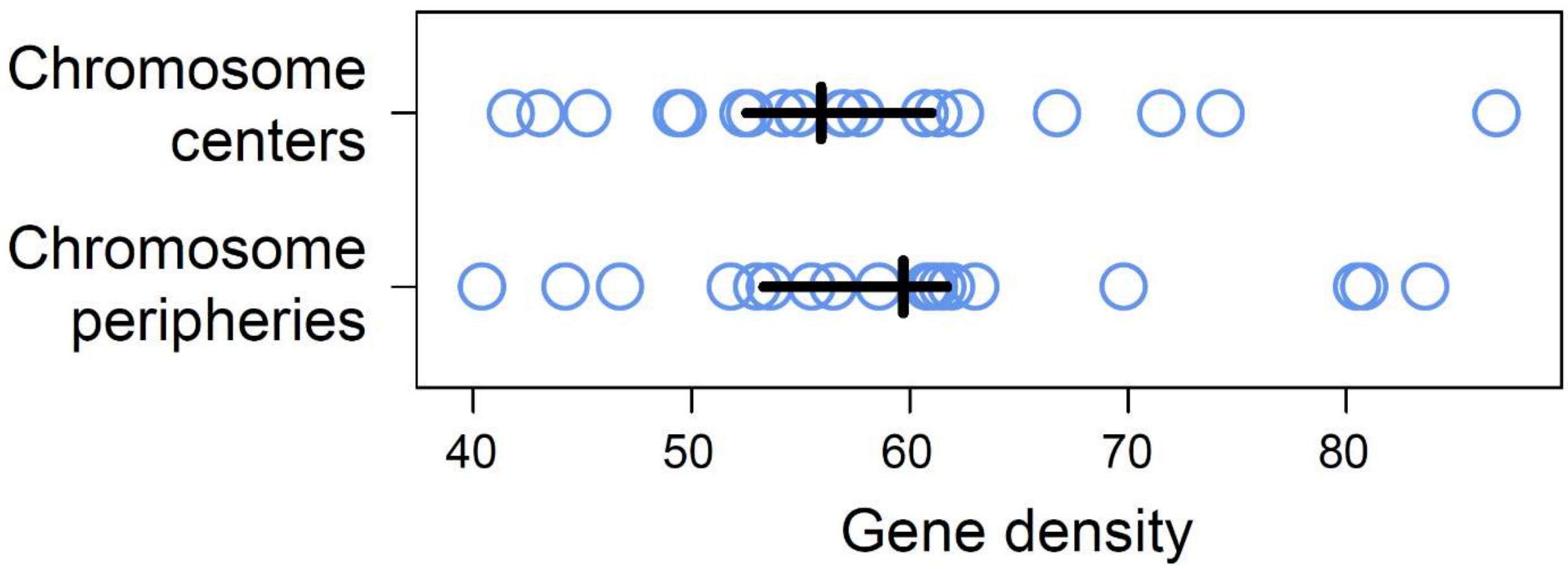

Figure S2. Gene density in the center versus peripheries of the threespine stickleback chromosomes, expressed as the number of genes per megabase. The chromosome peripheries are defined as the terminal 5 Mb on either side of each chromosome. The blue circles represent values for individual chromosomes, the vertical black lines indicate the median across these values, and the horizontal black lines show the 95% compatibility interval for this median based on 10,000 bootstrap resamples. The compatibility intervals are very similar, indicating no substantial difference in gene density between the two chromosome regions.

Figure S3

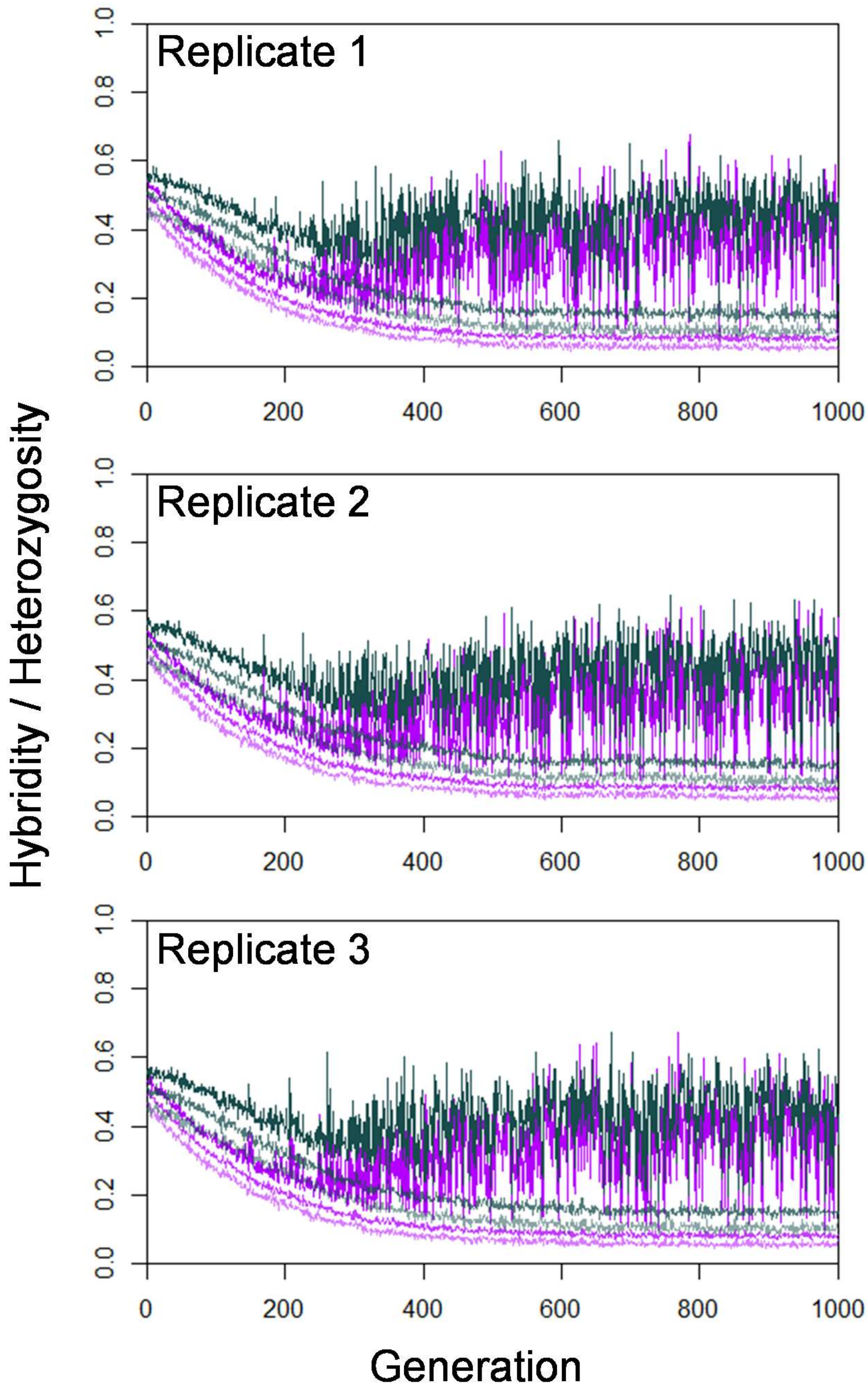

Figure S3. Establishment of migration-selection equilibrium during the simulations of lake-stream divergence with the stepping-stone model using ABC estimation optima for the *f* and *m* parameters. Shown are the 0.125, 0.5, and 0.875 quantiles (distinguished by increasing color intensities) for hybridity (purple) and heterozygosity (gray) across 40 individuals sampled from the first stream deme in the model in each generation. Results

are shown for three replicate simulation runs. Alleles are initially sampled at random with a probability of 0.5, hence the genetic composition is initially uniform across the entire model. After around 500 generations, the system reaches migration-selection equilibrium, as revealed by the divergence within this deme between lake-adapted immigrants and strongly admixed individuals, the latter captured by the top quantiles.

Figure S4

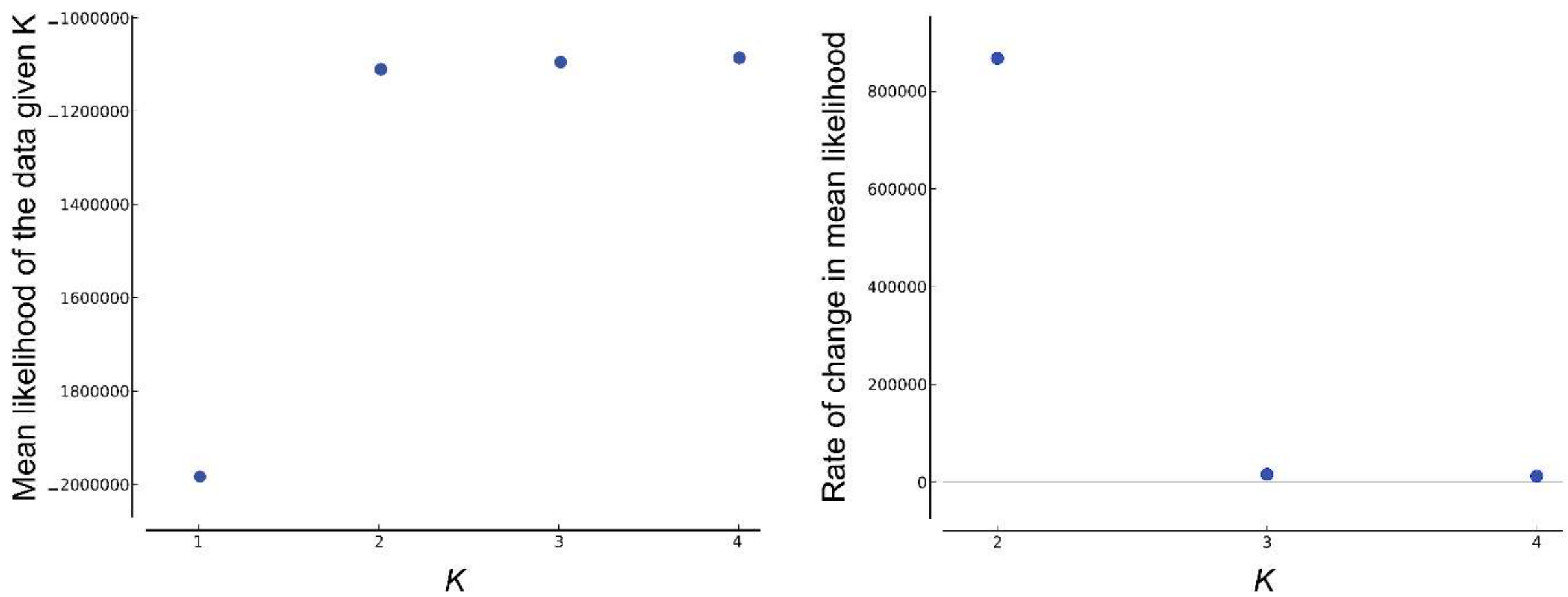

Figure S4. Analysis of population structure with the software STRUCTURE, based on the ecotype-distinctive SNPs and including all 100 individuals from the Misty lake-stream system. The left graphic shows the likelihood *L(K)* of the genomic data when assuming different numbers of populations (*K*), averaged across 20 replicate runs for each *K*. This demonstrates that assuming a single population across the Misty system is implausible, and that beyond *K* = 2, there is minimal gain in likelihood. The rate of change in likelihood *L'(K)* (right graph) thus clearly reveals two true populations (*K* = 2), as does the ∆*K* statistic derived from this metric (not shown).

Figure S5

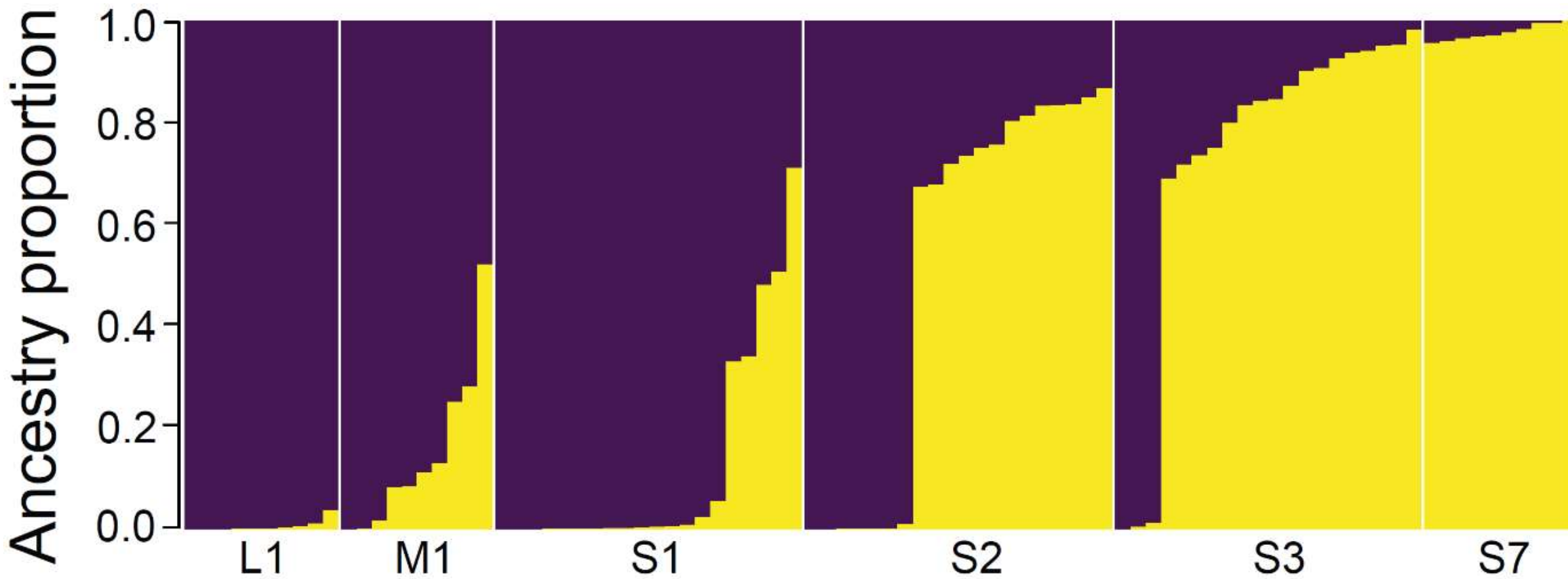

Figure S5. Global ancestry proportions as estimated by STRUCTURE. The graph follows the conventions of Figure 2B, except that the underlying markers are random SNPs, not ecotype-distinctive ones.

Figure S6

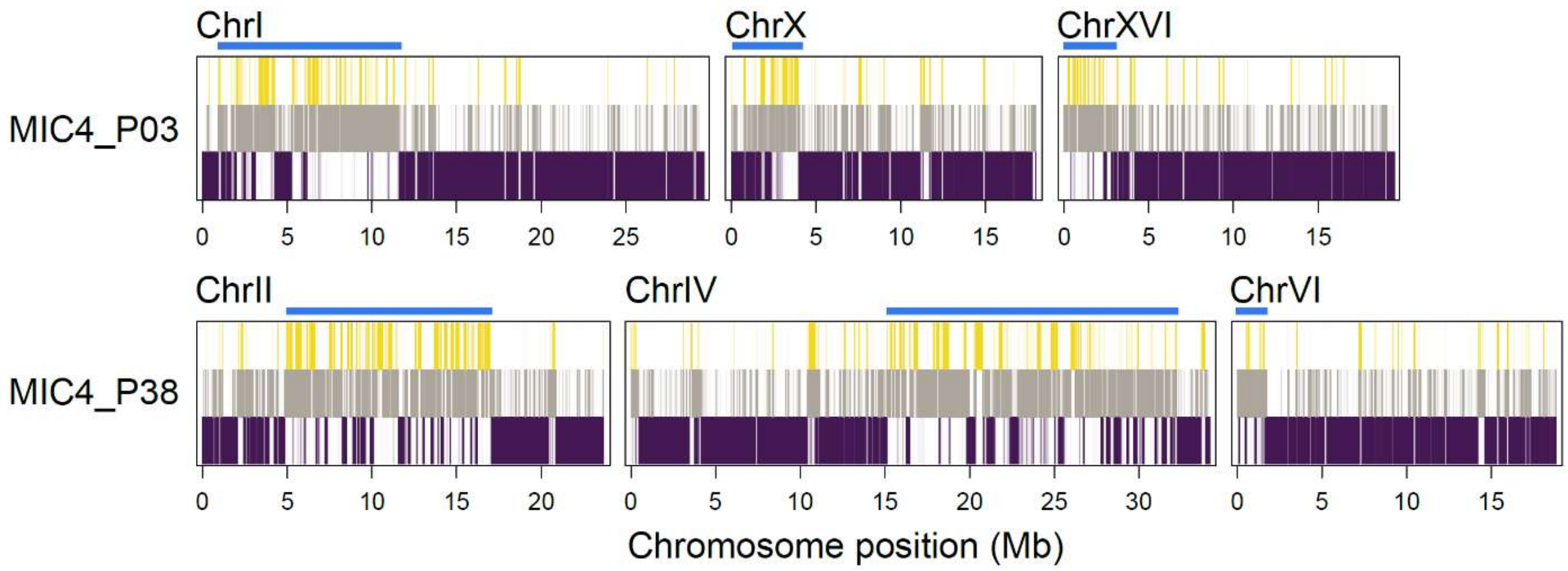

Figure S6. Two individuals from the first stream site (S1) showing distinctive tracts of alternative ancestry along some chromosomes. Specifically, while most chromosomes in these individuals exhibited predominantly homozygous lake ancestry (like in Figure 3B, top row), a few chromosomes displayed extended tracts of heterozygous ancestry (approximately indicated by horizontal blue bars), suggesting relatively recent backcrossing of dispersers from the lake with admixed local individuals.

Figure S7

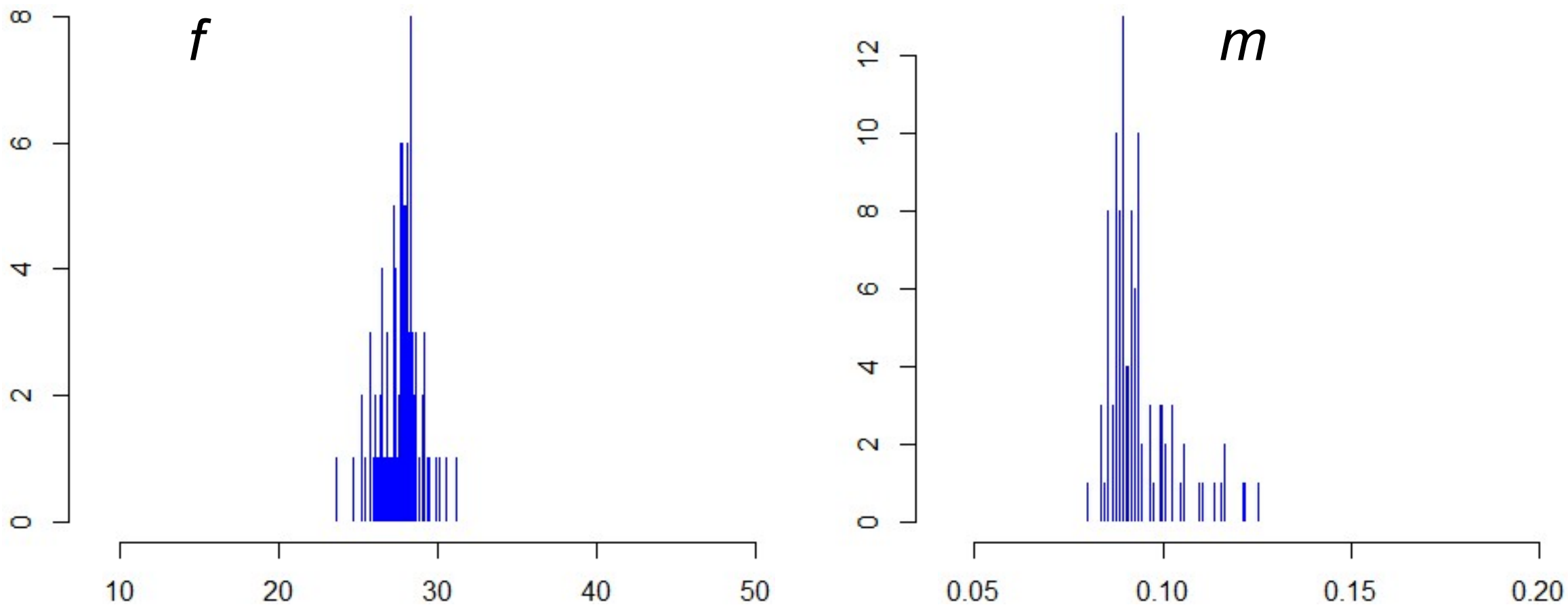

Figure S7. Distribution of Approximate Bayesian Computation (ABC) parameter estimates for the factor *f* determining the size of the lake deme relative to the size of the stream demes (*n* = 200), and for the proportion *m* of individuals dispersing in each generation from a given deme into each neighboring deme (except for the terminal demes, total emigration is thus 2*m*). The distributions are based on 100 replicate ABC estimation runs, all performed with hybridity and heterozygosity summary statistics from 300 simulations with values of *f* and *m* chosen at random. Shown are median estimates from each estimation run. The grand medians (27.8 and 0.091 for *f* and *m*) were taken as optima for further simulation.

Figure S8

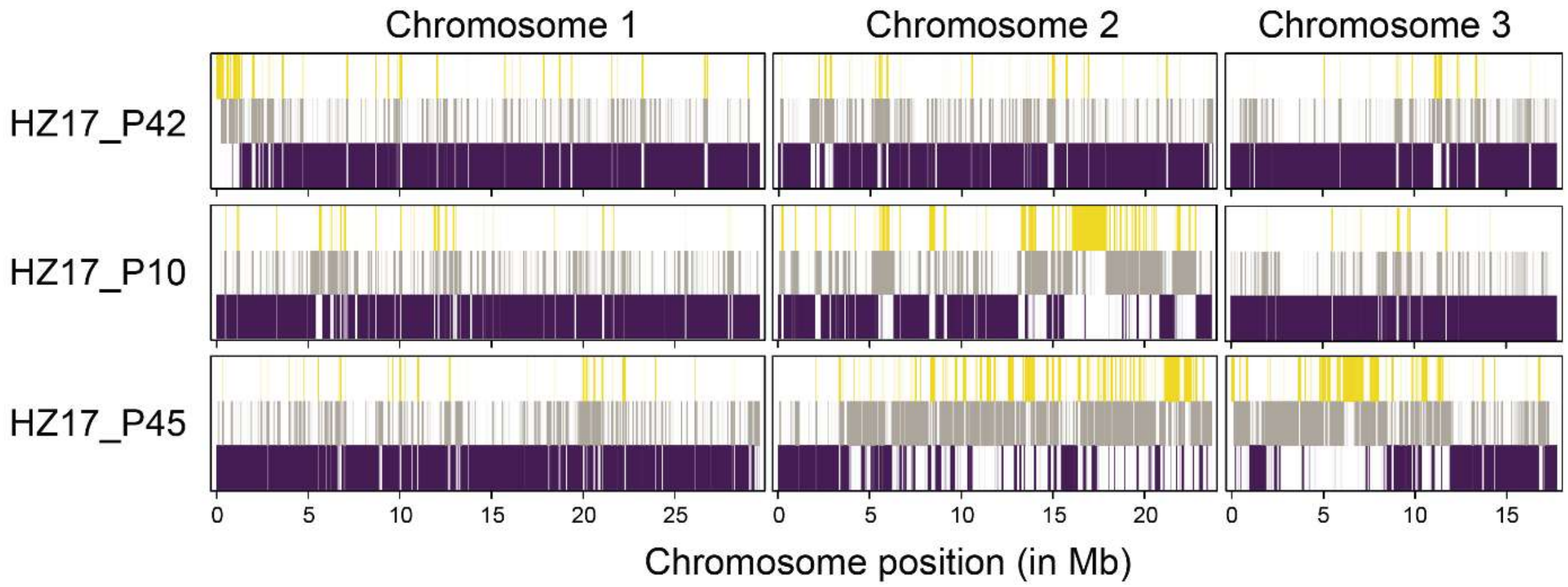

Figure S8. Local ancestry along three chromosomes for three exemplary marsh individuals collected in 2017. The selected individuals include the one with the lowest (0.057, top) and the highest (0.187, bottom) hybridity observed in this sample, plus an intermediate one (0.101). All graphing conventions correspond to those of Figure 3B.
